# β-Cyclodextrin-Based Inclusion Complexation Bridged Biodegradable Self-Assembly Macromolecular Micelle for the Delivery of Paclitaxel

**DOI:** 10.1371/journal.pone.0150877

**Published:** 2016-03-10

**Authors:** Yanzuo Chen, Yukun Huang, Dongdong Qin, Wenchao Liu, Chao Song, Kaiyan Lou, Wei Wang, Feng Gao

**Affiliations:** 1 Shanghai Key Laboratory of Functional Materials Chemistry, East China University of Science and Technology, Shanghai 200237, China; 2 Department of Pharmaceutics, School of Pharmacy, East China University of Science and Technology, Shanghai 200237, China; 3 Shanghai Key Laboratory of New Drug Design, East China University of Science and Technology, Shanghai 200237, China; 4 Shanghai Key Laboratory of Chemical Biology, and State Key Laboratory of Bioengineering Reactor, East China University of Science & Technology, Shanghai 200237, China; 5 Department of Chemistry and Chemical Biology, University of New Mexico, Albuquerque, NM 87131-0001, United States of America; University of Helsinki, FINLAND

## Abstract

In this study, a novel adamantanamine-paclitaxel (AD-PTX) incorporated oligochitosan- carboxymethyl-β-cyclodextrin (CSO-g-CM-β-CD) self-assembly macromolecular (CSO-g-CM-β-CD@AD-PTX) micelle was successfully prepared in water through sonication. The formed molecules were characterized by Fourier transform infrared spectroscopy, proton nuclear magnetic resonance (NMR) spectroscopy, two-dimensional NMR, elemental analysis, and liquid chromatography-mass spectrometry, while the correspondent micelles were characterized by dynamic light scattering and transmission electron microscopy. We showed that the macromolecular micelle contained a spherical core-shell structure with a diameter of 197.1 ± 3.3 nm and zeta potential of −19.1 ± 4.3 mV. The CSO-g-CM-β-CD@AD-PTX micelle exhibited a high drug-loading efficacy up to 31.3%, as well as a critical micelle concentration of 3.4 × 10^-7^ M, which indicated good stability. Additionally, the *in* vitro release profile of the CSO-g-CM-β-CD@AD-PTX micelle demonstrated a long-term release pattern, 63.1% of AD-PTX was released from the micelle during a 30-day period. Moreover, the CSO-g-CM-β-CD@AD-PTX micelle displayed cytotoxicity at a sub-μM scale similar to PTX in U87 MG cells, and CSO-g-CM-β-CD exhibited a good safety profile by not manifesting significant toxicity at concentrations up to 100 μM. These results indicated that β-CD-based inclusion complexation resulting in biodegradable self-assembled macromolecular micelles can be utilized as nanocarrier, and may provide a promising platform for drug delivery in the future medical applications.

## Introduction

Nanotechnology advancement has significantly impacted the field of drug delivery. Hence, a range of formulations for the delivery of anticancer drugs exhibiting poor solubility have been developed based on nanoparticles, liposomes, and polymeric micelles. [1–3] In particular, polymeric micelles have attracted significant interest as a novel drug delivery system[4] that possesses a variety of advantages, including high solubility, sustained drug-release profiles, and easy surface functionalization which allow polymeric micelles being employed as a carrier for anticancer drugs exhibiting poor solubility. [5]

New drug-loading techniques could provide alternative solutions to overcome problems, such as *in vivo* instability, low drug-loading capability, and potential safety issues associated with organic solvent residues.[6, 7] Utilizing non-covalent interactions in water such as ionic interaction[8] or stereocomplexation[9] for construction of polymeric micelles has constituted one approach to fixing these problems. β-Cyclodextrin (CD), an important biodegradable water-soluble host molecule in supramolecular chemistry, has attracted much attention due to its rigid, well-defined ring structure and hydrophobic inner cavity.[10] β-CD is capable of forming a stable inclusion complex with adamantanamine (AD) in water that is based on hydrophobic interactions that occur as the AD molecule inserts into the inner cavity of CD to form a host-guest pair with a high association constant of nearly 1 × 10^5^ M^−1^.[11] This host-guest interaction could be used to construct a more complex system given AD pre-conjugation with other bioactive molecules, such as a bulky hydrophobic drug. In addition to the initial self-assembly and formation of the host-guest inclusion complex between AD and β-CD, the bulky hydrophobic drug linked to AD might aggregate upon exposure to water to form the hydrophobic core, enabling further micelle self-assembly. Besides β-CD, oligochitosan (CSO) is a biodegradable and biocompatible water-soluble hydrolysate of chitin used in this work, and has also been revealed to form micelle structures for the encapsulation of a variety of anticancer drugs.[12–14]

Paclitaxel (PTX), a natural hydrophobic diterpenoid isolated from the bark of the pacific yew tree, displayed potent anticancer activity by promoting assembly and stability of microtubules, inhibiting mitosis, and inducing cellular apoptosis.[2, 15] PTX has been widely used as a first-line drug in the treatment of a number of solid tumors, including breast, ovarian, and prostate cancer.[16, 17] However, its therapeutic application has been severely limited due to its unfavorable pharmacokinetic properties such as poor solubility and reduced cell-penetration due to P-glycoprotein transporter-mediated active efflux.[14, 18, 19]

To date, some of the complex self-assembly macromolecular consisting of dual-stage assembly processes for drug delivery have been reported. [20, 21] For example, poly-ethylenimine-α-cyclodextrin (PEI-CD) and 2-adamantanamine-conjugated paclitaxel (AD-PTX) were utilized for the co-delivery PTX and survivin shRNA-encoding plasmids to SKOV-3 cells grafted mice, [4] which resulted in a significant improvement in tumor-growth suppression relative to other single-agent therapeutic treatments used. Additionally, the same research group also investigated another core-shell-based nano-assembly through host-guest interactions between β-CD and the benzyl group of poly(β-benzyl-L-aspartic) for co-delivery of dexamethasone and therapeutic pDNA. The resulting self-assembled macromolecule enhanced the co-delivery transfection efficiency; however, the *in vitro* stability and *in vivo* safety were not illustrated thoroughly and the relatively low drug loading efficacy, poor *in vivo* stability of genes and PEI-related safety issues associated with of these formulations also might limit their therapeutic effects and long-term applications.[22] Besides, although the therapeutic efficiency of some complex self-assembly macromolecular loaded with certain anticancer drugs such as doxorubicin and PTX have been investigated, the role of AD-PTX played alone in the drug delivery system has not been thoroughly illustrated in the papers already published to our knowledge.

**Fig 1:**
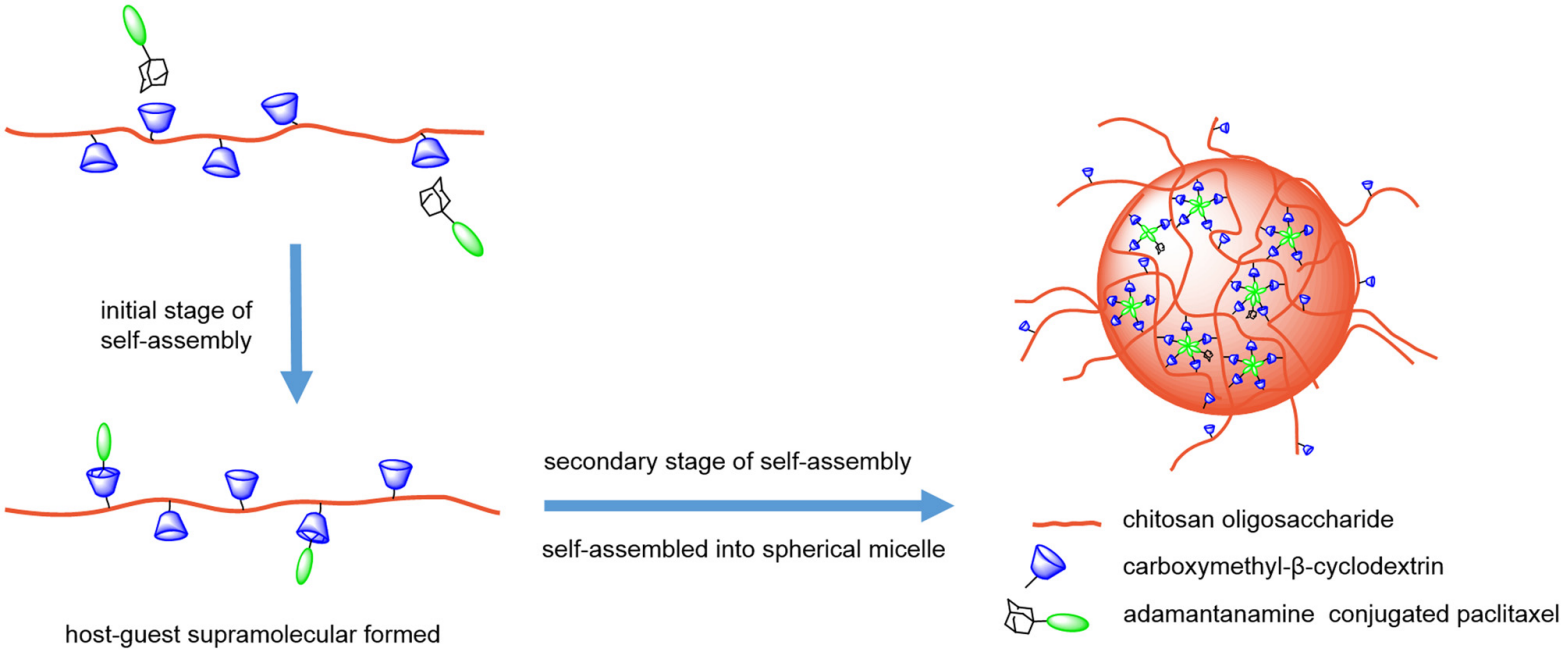
Schematic illustration of the preparation of the CSO-g-CM-β-CD@AD-PTX micelle.

In this study, we reported a macromolecular micelle drug-delivery system with high drug-loading efficacy and good stability based on the formation of inclusion complexes between the guest drug molecule adamantanamine-paclitaxel (AD-PTX) and polymeric host oligochitosan-carboxymethyl-β-cyclodextrin (CSO-g-CM-β-CD). AD-PTX was obtained through the conjugation between PTX and AD. The polymeric host CSO-g-CM-β-CD was synthesized from carboxymethyl-β-cyclodextrin (CM-β-CD) and CSO under the amide-bond formation conditions. In our design, CM-β-CD provide the host site, and the adamantanamine moiety in AD-PTX served as the guest to be inserted into the inner hydrophobic pocket of CM-β-CD. CSO also provided the hydrophilic backbone, as well as sites for further modification. The schematic representation of the macromolecular self-assembly process to form the micelle is illustrated in Fig 1. We suggested that the macromolecular micelle system was formed by consecutive two-stage self-assembly process in aqueous solution by sonication, with the first stage involved the host-guest interaction between AD-PTX and CM-β-CD units in the CSO backbone and the second stage, resulting in aggregation and cross-linking of the bulky hydrophobic portion of PTX to form spherical macromolecular micelle.

## Experimental section

### Materials

Oligochitosan (Mw = 5 kDa, 90.0% deacetylation degree) was obtained from Aoxing Co. Ltd. (Hangzhou, Zhejiang, China), carboxymethyl-β-cyclodextrin was purchased from Zhiyuan Biotech Co., Ltd. (Shandong, China). Paclitaxel was provided by Xi’an Sanjiang Bio-Engineering Co., Ltd. (Xi’an, Shanxi, China) and free PTX solution was prepared according to the commercial formulation of Taxol^®^. Adamantanamine, 1-ethyl-3-(3-dimethylaminopropyl)carbodiimide hydrochloride, succinic anhydride, and 4-dimethylaminopyridine are obtained from Sigma (St. Louis, MO, USA). Purified deionized water was prepared by the Milli-Q plus system (Millipore Co., Billerica, MA, USA). All other reagents and chemicals were analytical grade.

3-(4,5-dimethyl-thiazol-2-yl)-2,5-diphenyl-tetrazolium bromide (MTT) was purchased from Sigma (St. Louis, MO, USA). Penicillin—streptomycin, Dulbecco’s Modified Eagle Medium (DMEM), fetal bovine serum (FBS) and, 0.25% (w/v) trypsine 0.03% (w/v) EDTA solution were purchased from Gibco BRL (Gaithersberg, MD, USA).

Human primary glioblastoma cell line U87 MG cells were obtained from Cell Institute of Chinese Academy of Sciences (Shanghai, China). Culture plates and dishes were obtained from Corning Inc. (NY, USA). The cells were cultured in DMEM medium, supplemented with 10% FBS, 100 IU/mL penicillin and 100 mg/mL streptomycin sulfate. The cells were cultured in incubators which were maintained at 37°C with 5% CO_2_ under fully humidified conditions.

### CSO-g-CM-β-CD synthesis

CSO-g-CM-β-CD was synthesized as described, with minor modifications.[23] Briefly, CM-β-CD (4.02 g) and 1-ethyl-3-(3-dimethylaminopropyl)carbodiimide (EDC, 0.96 g) were dissolved in 10 mL water, followed by addition of CSO (0.87) g. The reaction mixture was stirred for 48 h at 0–5°C, and dialyzed in cellulose-membrane tubing for 9 days with 12 exchanges of dialysis medium (water). The final solution was lyophilized and stored at –20°C until use.

### CSO-g-CM-β-CD Characterization

Fourier transform infrared (FT-IR) analysis was performed on a Nicolet 6700 FT-IR spectrometer (Thermo Scientific, Waltham, MA, USA). CSO and CSO-g-CM-β-CD were mixed and ground with spectroscopic potassium bromide, then compressed into a transparent disk before being placed in the sample cell. All spectra were scanned over the range 500–4000 cm^-1^ at a resolution >0.09 cm^-1^. Proton nuclear magnetic resonance (^1^H-NMR) spectra of CSO and CSO-g-CM-β-CD were recorded on a Bruker AVANCE 400 spectrometer (Bruker, Billerica, MA, USA) at 400 MHz using D_2_O as the solvent. Elemental analysis were performed in a Euro EA 3000 Series Elemental Analyzer (Eurovector, Milan, Italy) to determinate the degree of substitution, which was calculated as follows:

Degree of substitution=(14−(249 × N%))/(1173 × N%) (1)

where *N%* represents the nitrogen content of the samples.[24]

### AD-PTX synthesis

#### 2’-succinyl-PTX synthesis

PTX (1.17 g), succinic anhydride (0.07 g) and DMAP (0.04 g) were dissolved in 5.0 mL of anhydrous pyridine and stirred for 3 h at room temperature. Succinyl-PTX was purified by silica gel column chromatography and eluted with dichloromethane and acetone mixture (7/1, v/v).

#### AD-PTX synthesis

2’-succinyl-PTX (0.18 g), AD (0.40 g), 1-ethyl-3-(3-dimethylaminopropyl)carbodiimide hydrochloride (EDCI 0.46 g) and DMAP (0.27 g) were added in 30.0 mL dichloromethane, and the reaction was carried out overnight. The AD-PTX was purified by silica gel column chromatography eluted with a mixture of dichloromethane and acetone (10/1, v/v).

### AD-PTX characterization

Liquid chromatography mass spectrometry (LC-MS) was performed using a Waters SQ Detector 2 Single-quadrupole mass spectrometer (Waters; Milford, MA, USA) coupled to a Waters E2965 high-performance liquid chromatography (HPLC) system (Waters). AD-PTX was separated with other residues on a Zorbax XDB C_18_ column [4.6 mm × 250 mm, 5 μm (Agilent; Santa Clara, CA, USA)]. The mobile phase composition was a mixture of water/methanol (20:80, v/v) at a flow rate of 0.5 mL/min, and an injection volume of 20 μL. The mass spectrometer was operated using electrospray ionization (ESI) with an ion-spray voltage of +3800 V. The positive ion multiple-reaction-monitoring mode analysis was performed with nitrogen was employed as the collision gas. All HPLC and MS parameters were controlled by Masslynx software version 4.0 (Waters). The ^1^H-NMR spectrum of AD, PTX, and AD-PTX were recorded on a Bruker AVANCE 400 spectrometer (Bruker) at 400 MHz using DMSO-d_6_ as the solvent.

### Preparation of the CSO-g-CM-β-CD@AD-PTX micelle

CSO-g-CM-β-CD (80 mg) was dissolved in 2 mL phosphate buffer solution (pH = 7.4) and added dropwise into a solution of AD-PTX (60.0 mg) in DMSO (1.0 mL). The mixture was stirred for 8 h, and transferred into a dialysis membrane (molecular weight cut-off = 3.5 kDa, Yuanye Bio Co., Ltd, Shanghai, China) and dialyzed against the deionized water for 24 h to obtain the macromolecular solution, which was then filtered and lyophilized for further use.

The CSO-g-CM-β-CD@AD-PTX micelle was obtained via ultrasonication. Briefly, different amount of CSO-g-CM-β-CD/AD-PTX were dissolved in 3.0 mL phosphate buffer solution (pH 5–8). The solution was then calibrated to 3.0 mL and then sonicated using a probe sonicator (BILON92-11DL ultrasonic cell disruptor) at 100 W for 2 min in cycles of 1-s followed by 1.5-s of pauses.

### Characterization of the CSO-g-CM-β-CD@AD-PTX micelle

To determine the AD-PTX loading efficiency, macromolecular CSO-g-CM-β-CD@AD-PTX was dissolved in phosphate-buffered saline (PBS; pH 7.4) containing 0.5% Tween and then mixed with an excess amount of AD, followed by sonication to displace the AD-PTX from the inner cavities of CM-β-CD. Then, AD-PTX solution were mixed with the mobile phase and AD-PTX concentration was determined by HPLC. The AD-PTX loading efficiency in CSO-g-CM-β-CD@AD-PTX was calculated as follow:

$$\text{LE\%}=\frac{weight\;of\;AD-PTX\;in\;\text{macromolecular}}{weight\;of\;the\;feeding\;polymer\;and\;AD-PTX}\;\times \;100\%$$ (2)

To confirm the formation of macromolecular, the two-dimensional (2D) nuclear Overhauser spectroscopy (NOESY) spectrum of CSO-g-CM-β-CD@AD-PTX in D_2_O were recorded, and the corresponding cross peaks were verified.

The particle size and zeta potential of the CSO-g-CM-β-CD@AD-PTX micelles were determined using dynamic light scattering (DLS; Zetasizer Nano ZS, Malvern, UK), and the concentration of CSO-g-CM-β-CD@AD-PTX was kept at 0.5 mg/mL. The solutions were lyophilized to obtain dry micelles for transmission electron microscope (TEM) [JEM-2010; JEOL, Tokyo, Japan]. The sample was stained with 2% (w/v) phosphotungstic acid and then dropped on a copper grid.

### Measurement of critical micelle concentration (CMC)

The CMC was determined by fluorescence spectroscopy using pyrene as the fluorescence probe.[19]. Briefly, 5.0 mL of the micelle solutions with concentrations ranging from 1.0×10^−4^ to 0.4 mg/ml were added separately into volumetric flasks containing pyrene (final concentration is 297 μM). The excitation spectra were recorded from 350 to 450 nm and the λ_em_ = 395 nm. The fluorescent intensity was recorded at λ_ex_ = 373 and 385 nm, and the λ_em_ = 395 nm. The alteration in the ratio of intensity I_373_/I_385_ of pyrene with concentration of copolymer was used to calculate the CMC.

### *In vitro* stability

The optimal formulation of CSO-g-CM-β-CD@AD-PTX was lyophilized and stored at 4°C for 30 days in order to investigate its storage stability. Particle size was monitored at predetermined time points during the storage period.

### *In vitro* release

AD-PTX release from the CSO-g-CM-β-CD@AD-PTX micelle was monitored by dialysis. The macromolecular micelle (5.0 mg) and 2.0 mL PBS (pH = 7.4) were introduced into a dialysis tubing (molecular weight cut-off = 3.5 kDa, Yuanye Bio Co., Ltd, Shanghai, China) and submerged into 20.0 mL PBS at 37°C with stirring at 100 rpm for 30 days. At each appropriate time point (day 1–7, 14, 21 and 30), 0.5 mL aliquots were withdrawn and replaced with an equal volume of fresh medium. Each sample was supplemented with 1.0 mL acetonitrile and sonicated before AD-PTX concentration was determined by HPLC.

### *In vitro* cell-viability assay

A tetrazolium salt (MTT) assay was used to determine the cell viability of CSO-g-CM-β-CD@AD-PTX micelles. The U87 MG cells were seeded in 96-well plates at a density of 5 ×10^3^ cells/well for 24 h. Then the growth medium was removed and cells were incubated in media containing PTX, AD-PTX, CSO-g-CM-β-CD, AD, or CSO-g-CM-β-CD@AD-PTX micelle. After 72-h incubation, 180 μL of fresh growth medium and 20 μL of MTT (5 mg/mL) solution were added to each well. The plate was incubated for another 4 h, 200 μL DMSO added to dissolve the purple formazan crystals, and the plate was shaken vigorously before measurement. The absorbance at 490 nm of each well was measured by a microplate reader (TecanSafire 2; Tecan Group Ltd., Männedorf, Switzerland).

### Statistical analysis

All experiments were performed at least three times and expressed as means ± SD. Data were analyzed for statistical significance using Student’s t-test. P < 0.05 was considered statistically significant.

## Result and Discussion

### CSO-g-CM-β-CD synthesis and characterization

**Fig 2:**
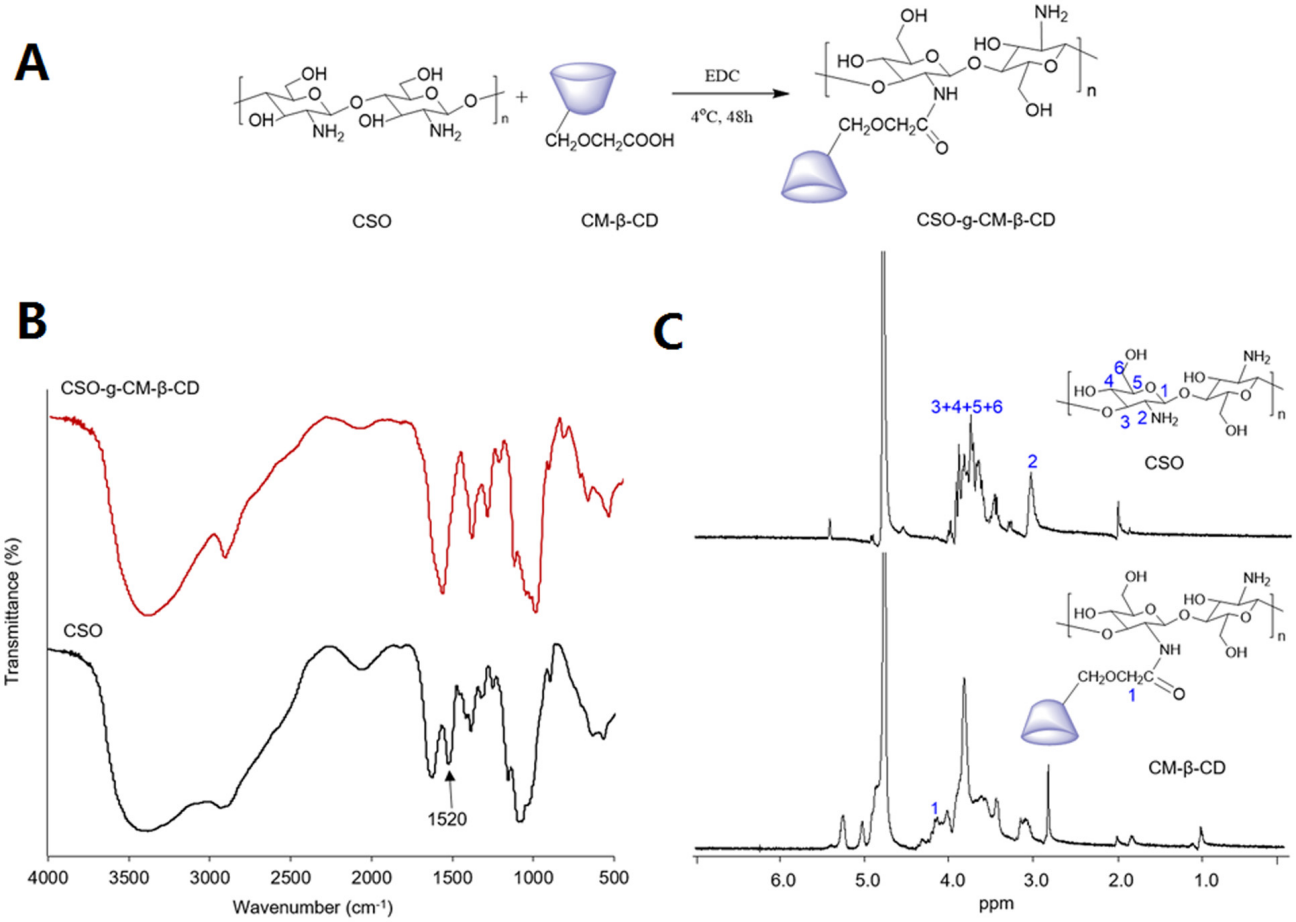
CSO-g-CM-β-CD synthesis and characterization. Synthetic route of CSO-g-CM-β-CD (A); FT-IR spectra of CSO-g-CM-β-CD and CSO (B) and ^1^H-NMR spectra of CSO and CSO-g-CM-β-CD in D_2_O (C).

CSO-g-CM-β-CD synthesis is outlined in Fig 2A, and the obtained CSO-g-CM-β-CD was characterized by FT-IR and ^1^H-NMR spectra. The FT-IR spectra of CSO-g-CM-β-CD (Fig 2B), indicated that the N-H in-plane bending vibration of the amine group in CSO at 1520 cm^-1^ had disappeared, signifying amine group conversion. The ^1^H-NMR spectra (Fig 2C) showed peaks from 3.0–3.9 ppm to be the protons from the CSO units. The emergence of characteristic peaks from units of CM-β-CD at 5.0 ppm and 4.3 ppm indicated the successful grafting of CM-β-CD to CSO, and forming CSO-g-CM-β-CD. The CSO-g-CM-β-CD substitution degree calculated by elemental analysis was 19.9 ± 0.1%.

### AD-PTX synthesis and characterization

**Fig 3:**
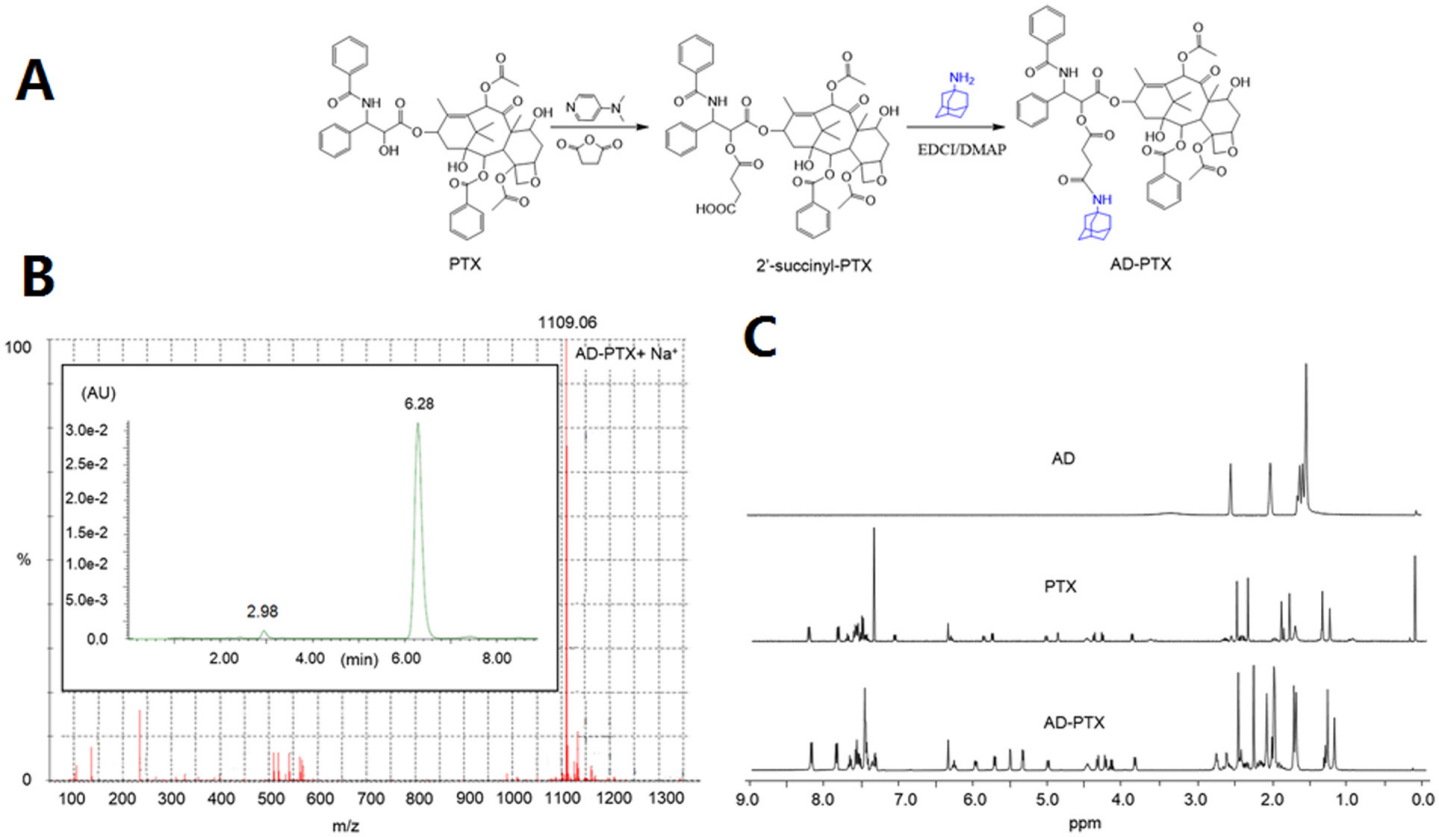
AD-PTX synthesis and characterization. Synthetic route of AD-PTX (A); LC-MS spectra of AD-PTX (B) and ^1^H-NMR spectra of AD, PTX and AD-PTX in DMSO-d_6_ (C).

AD-PTX synthesis is outlined in Fig 3A. AD-PTX was successfully synthesized by conjugation of activated 2'-succinyl-PTX with the amine group of AD, with the purity of the formed structure subsequently verified by HPLC-MS analysis (Fig 3B). The AD-PTX and PTX residues were well-separated at retention time at 6.28 min and 2.98 min, respectively, and displayed good resolution. The ESI mass spectra of AD-PTX exhibited a sharp peak at *m/z* 1109.96, which corresponded to the [M+Na^+^] peak calculated as 1109.45. Moreover, the ^1^H-NMR spectra of AD-PTX (Fig 3C) showed both characteristic peaks of PTX at *δ* 1.0–2.0 ppm and *δ* 7.0–8.0 ppm assigned to the methyl and phenyl groups, respectively, as well as characteristic peaks of adamantyl group of AD at *δ* 1.5–2.0 ppm. These results indicated the successful conjugation of AD to PTX.

### Characterization and properties of the CSO-g-CM-β-CD@AD-PTX micelle

**Fig 4:**
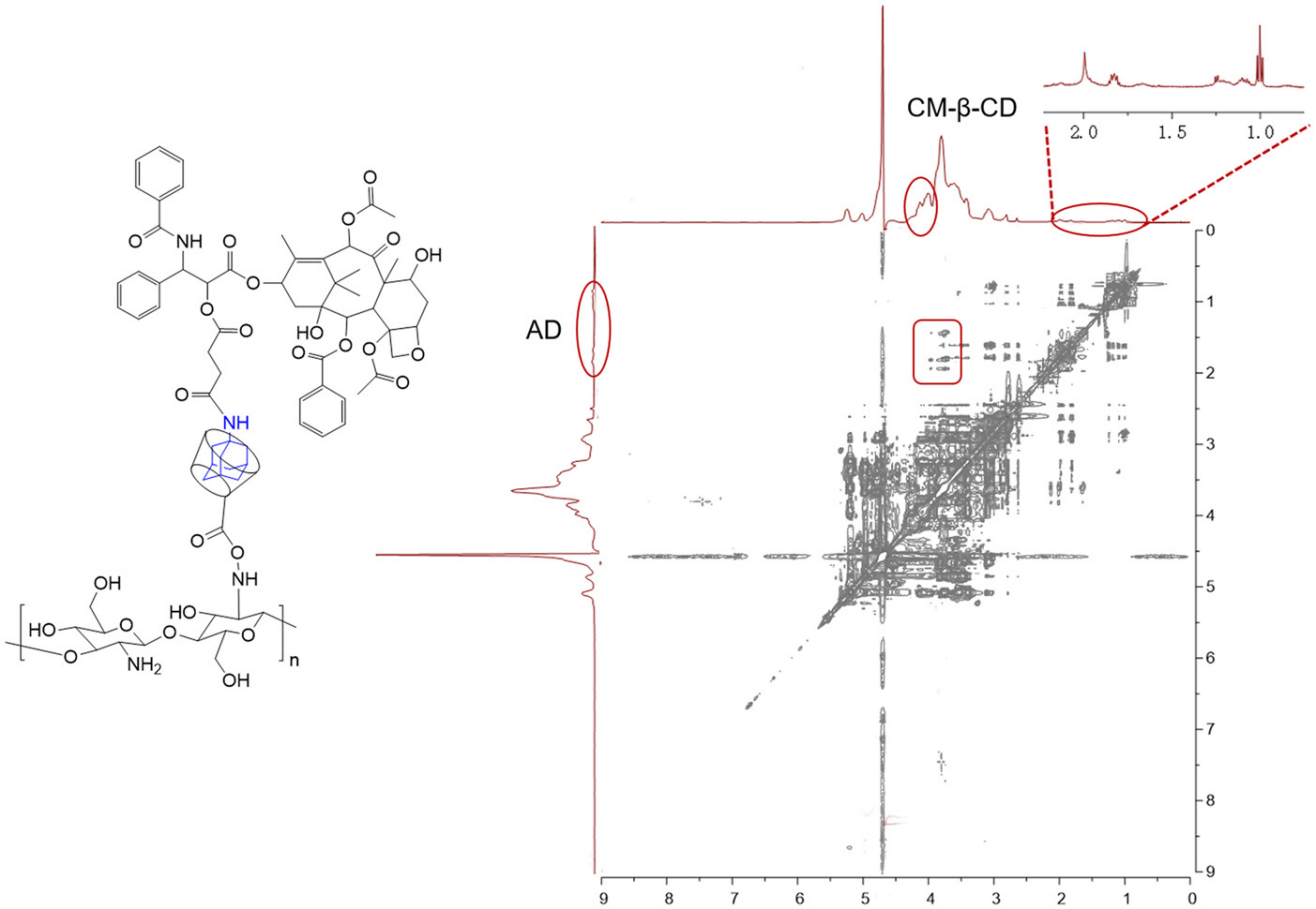
2D-NOESY NMR spectrum of CSO-g-CM-β-CD@AD-PTX in D_2_O.

Following replacement with excess AD, free AD-PTX was detected in the media, and the AD-PTX- loading efficiency of CSO-g-CM-β-CD@AD-PTX was determined as 31.3 ± 0.7% by HPLC. The 2D-NMR (NOESY) spectrum (Fig 4) was used to confirm the existence of host-guest interactions in the formation of CSO-g-CM-β-CD@AD-PTX. The cross peaks between the inner protons of CM-β-CD and the protons on the adamantyl moiety of the AD-PTX indicated the formation of CSO-g-CM-β-CD@AD-PTX macromolecules.

For nanoparticle-based drug delivery systems, particle size is an essential factor in the fate of the loaded drug *in vitro* and *in vivo*.[25] To optimize the particle size of the CSO-g-CM-β-CD@AD-PTX micelle during the sonication process, we investigated the effects of variations in pH on the particle size distribution and zeta potential of the macromolecular micelle (Table 1). The pH had more influence on the zeta potential of the macromolecular micelle than the particle size, which could be explained by pH-induced surface charge alternations due to deprotonation of the ammonium ion to amine group and the carboxyl acid group of CM-β-CD to carboxyl anion following pH elevation. As the pH reached 7, the size of the macromolecular micelle was stabilized at 197.1 ± 3.3 nm, which was within the acknowledged suitable range for achieving enhanced permeability and retention effects to facilitate passive targeting of unmodified nanoparticles to tumor site.[26, 27] Additionally, the negative charged out layer of the loaded macromolecular micelle could facilitate avoidance of rapid blood clearance initiated by the mononuclear phagocytic system, leading to a relatively extended circulation time *in vivo*.[22]

**Table 1:** The influence of pH on CSO-g-CM-β-CD@AD-PTX micelle formation.

pH	Micelle size (nm)	Zeta potential (mV)	PI^a^
5.0	281.1 ± 3.2	-7.5 ± 0.6	0.23
6.0	237.8 ± 5.0	-10.6 ± 0.6	0.25
7.0	197.1 ± 3.3	-19.1 ± 4.3	0.16
8.0	229.6 ± 2.2	-21.4 ± 4.1	0.39

^a^ PI: polydispersity index.

Data were presented as mean ± SD (n = 3).

**Fig 5:**
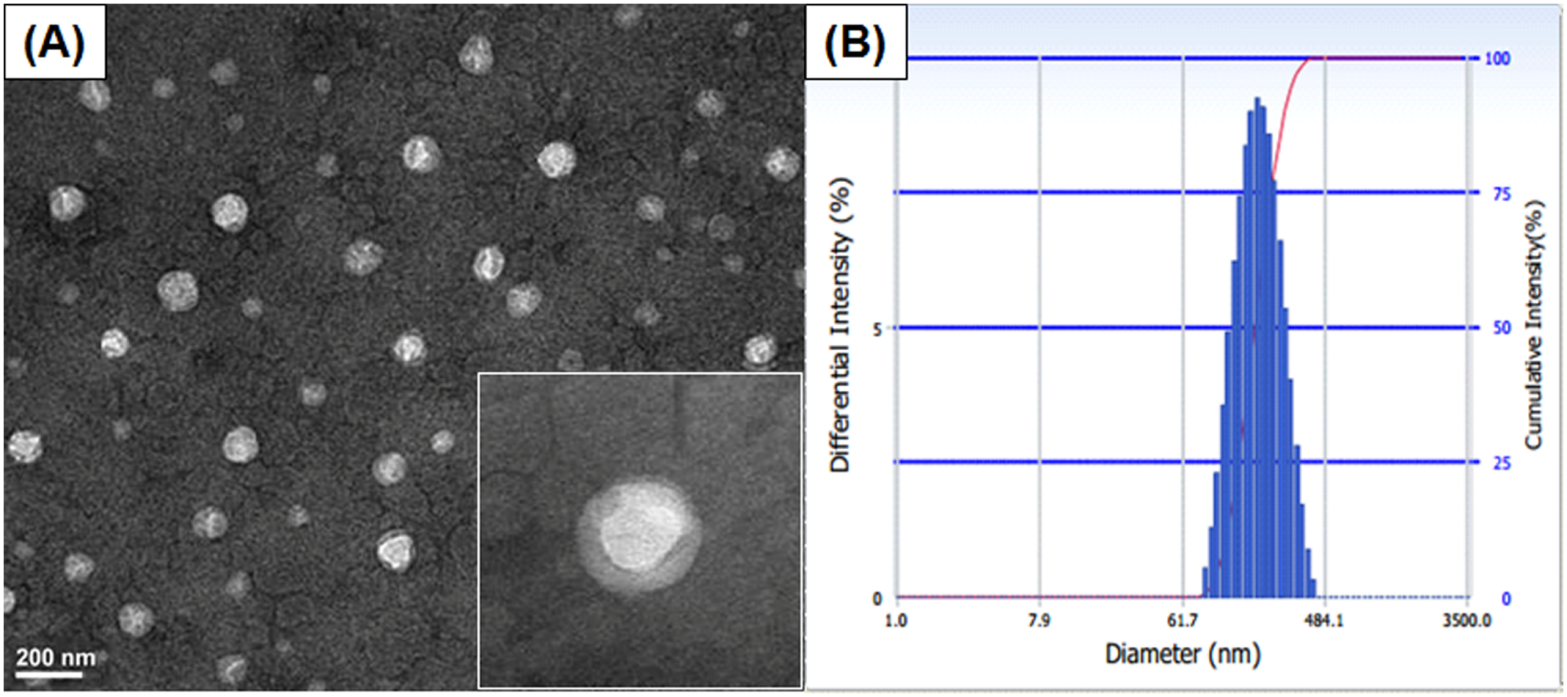
Characterization of CSO-g-CM-β-CD@AD-PTX micellar system. TEM images (A) and size distribution of CSO-g-CM-β-CD@AD-PTX micelles (B).

The formation of the macromolecular micelle was further confirmed by TEM imaging (Fig 5A and 5B). The CSO-g-CM-β-CD/AD-PTX micelles appeared dispersed as spherical core-shell structures homogeneously distributed at an average size of ~160 nm. However, the average particle size determined from TEM was smaller than 197.1 ± 3.3 nm detected by DLS in solution (Fig 5C). This inconsistency was likely due to the difference in measurement conditions between the two methods. The larger value obtained from DLS may have indicated a layer of solvent shell, while anhydrous conditions was used in TEM imaging.

**Fig 6:**
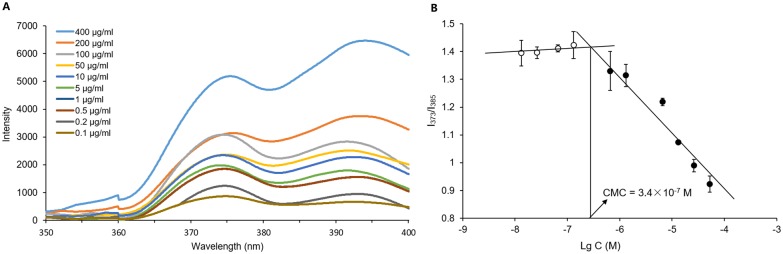
Fluorescence analysis. Fluorescence scanning analysis with pyrene (A) and critical micelle concentration of CSO-g-CM-β-CD@AD-PTX solution (B).

CSO-g-CM-β-CD@AD-PTX CMC was measured by fluorescence methods using pyrene as the fluorescent probe. The ratios of I_373_/I_385_ were plotted against the logarithm of polymer concentration, and CMC was represented as the concentration at the turning point (Fig 6). The obtained CMC from the plot was 3.4 × 10^−7^ M, suggesting that the CSO-g-CM-β-CD@AD-PTX micelle probably could be stable upon dilution for blood circulation.[28]

The *in vitro* stability was evaluated by the change of particle size in PBS after 30-day storage. During the period, the particle size of the macromolecular micelle changed slightly from 197.1 ± 3.3 nm to 208 ± 8.5 nm, indicating good stability.

### *In vitro* drug release profile of the CSO-g-CM-β-CD@AD-PTX micelle

**Fig 7:**
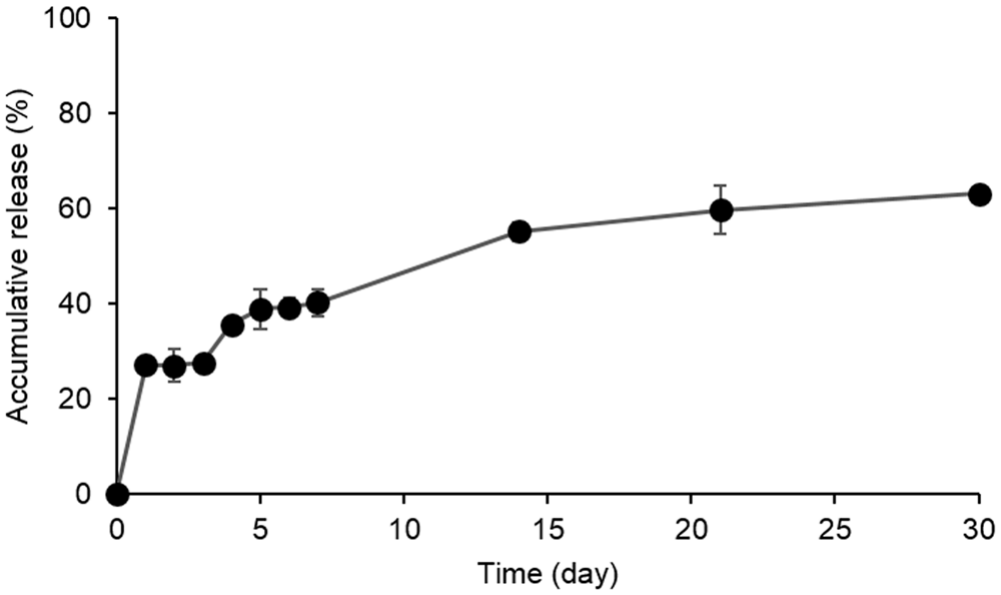
*In vitro* drug release of CSO-g-CM-β-CD@AD-PTX micelle in the PBS with pH = 7.4 (n = 3).

The *in vitro* drug-release profile of the CSO-g-CM-β-CD@AD-PTX micelle was examined at pH 7.4.(Fig 7) In the initial release phase, 27.2% of AD-PTX was released over the first day, which was induced by desorption of the surface-bound or loosely adsorbed drug. And the second release phase was progressively slower, with a total 63.1% released over 30 days. This could be the result of the drug diffusion through the micellar matrix and matrix erosion or degradation. These results indicated a good sustained release profile of the CSO-g-CM-β-CD@AD-PTX micelle.

### *In vitro* cell viability assay

**Fig 8:**
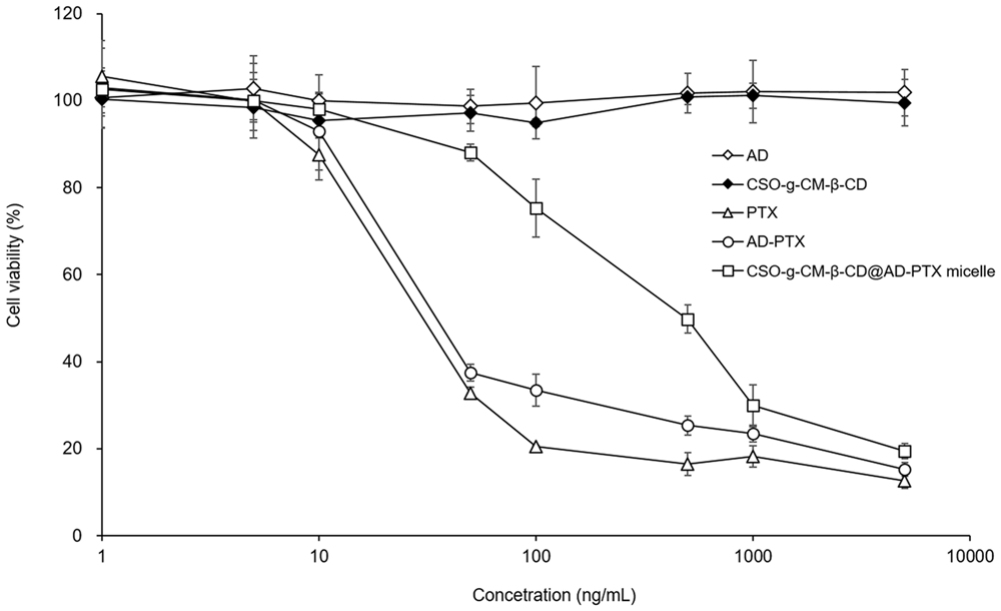
*In vitro* cell viability assay of different formulations on U87 MG cells for 72 h (n = 3).

To evaluate the antiproliferative efficacy of different PTX formulations on U87 MG cells, PTX, AD-PTX, AD, CSO-g-CM-β-CD, or CSO-g-CM-β-CD@AD-PTX micelles were incubated with U87 MG cells for 72 h, and the cell viability measured by MTT assay (Fig 8). The IC_50_ was 52.2 ± 8.1 nM, 106.5 ± 1.4 nM, and 129.9 ± 5.4 nM for PTX, AD-PTX and CSO-g-CM-β-CD@AD-PTX micelles, respectively. AD and CSO-g-CM-β-CD exhibited high IC_50_ values (> 100 μM), indicating excellent biocompatibility and biosafety as a potential drug carrier. Additionally, our results suggested all the PTX formulations have strong growth inhibition on U87 MG cells. The IC_50_ value of AD-PTX was only slightly higher than that of PTX, suggesting AD conjugation did not significantly affect the PTX efficacy. After loading, the IC_50_ values of the CSO-g-CM-β-CD@AD-PTX micelles was a slightly reduced, possibly due to low cytotoxicity of the carrier and the slow release profile of AD-PTX.

## Conclusion

In conclusion, we successfully prepared a novel macromolecular micelle drug-delivery system (CSO-g-CM-β-CD@AD-PTX micelle) with high drug-loading efficacy and good stability by sonication in water for the delivery of AD-PTX. The key to the successful self-assembly was the host-guest interactions between AD-PTX molecules and CM-β-CD units of CSO-g-CM-β-CD which then promoted the formation of inclusion complexes. The micelles had favorable negative surface charges necessary for extended circulation *in vivo*, as well as a core-shell structure with narrow size distribution (~190 nm by DLS determination) suitable for passive targeting to tumor sites. Additionally, CSO-g-CM-β-CD@AD-PTX micelle exhibited cytotoxicity levels in the sub-μM range, which was similar to PTX according to the MTT assay while unloaded CSO-g-CM-β-CD did not show any significant toxicity up to 100 μM, indicating an improved safety profile. Moreover, the macromolecular micelle exhibited a long-acting release profile, and the novel self-assembly method for micelle preparation reduced the concentration of organic solvent residues associated with many drug-related side effects. This method constitutes a versatile platform for combination treatments using multiple drugs. The results presented here suggest that our carrier is a promising platform for drug delivery in medical applications.
